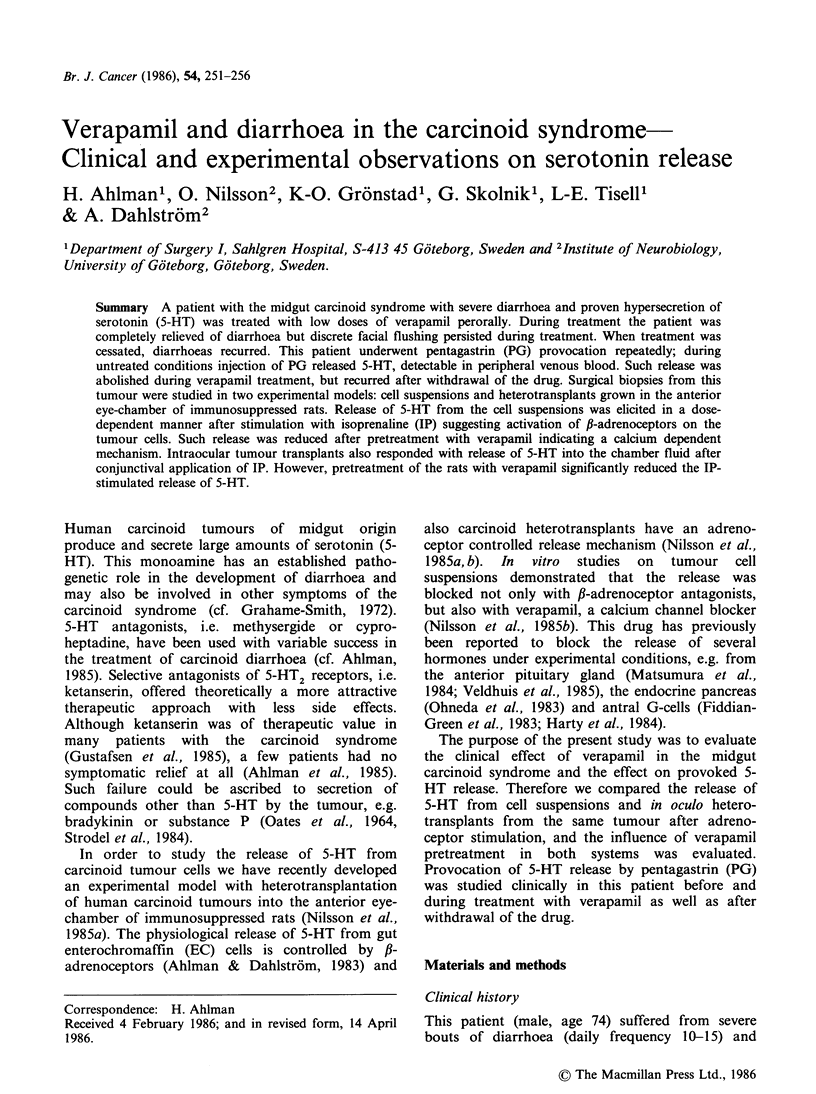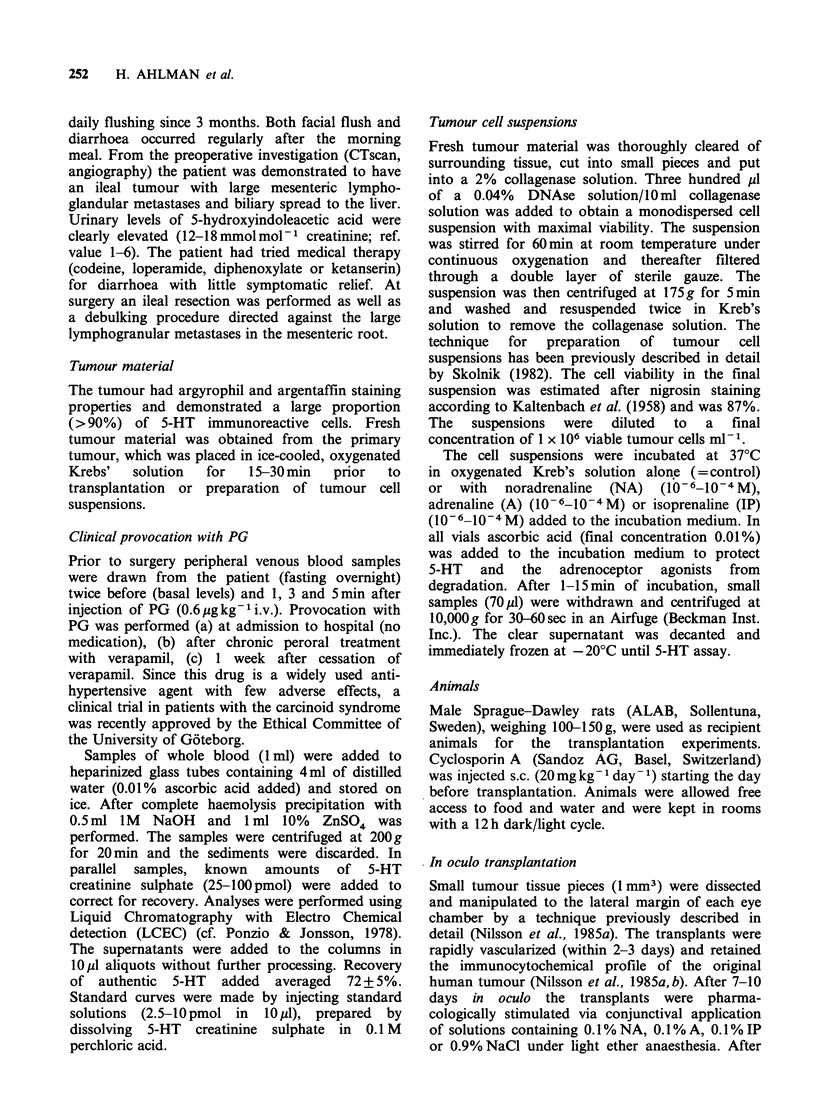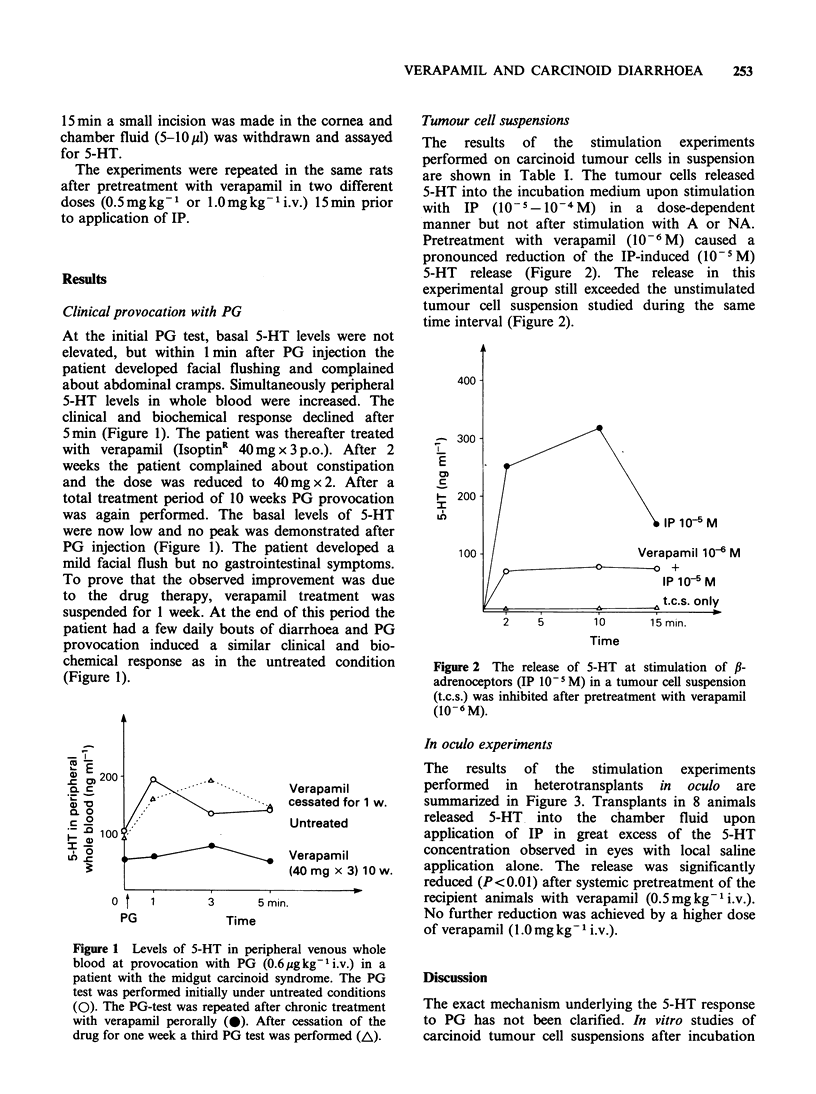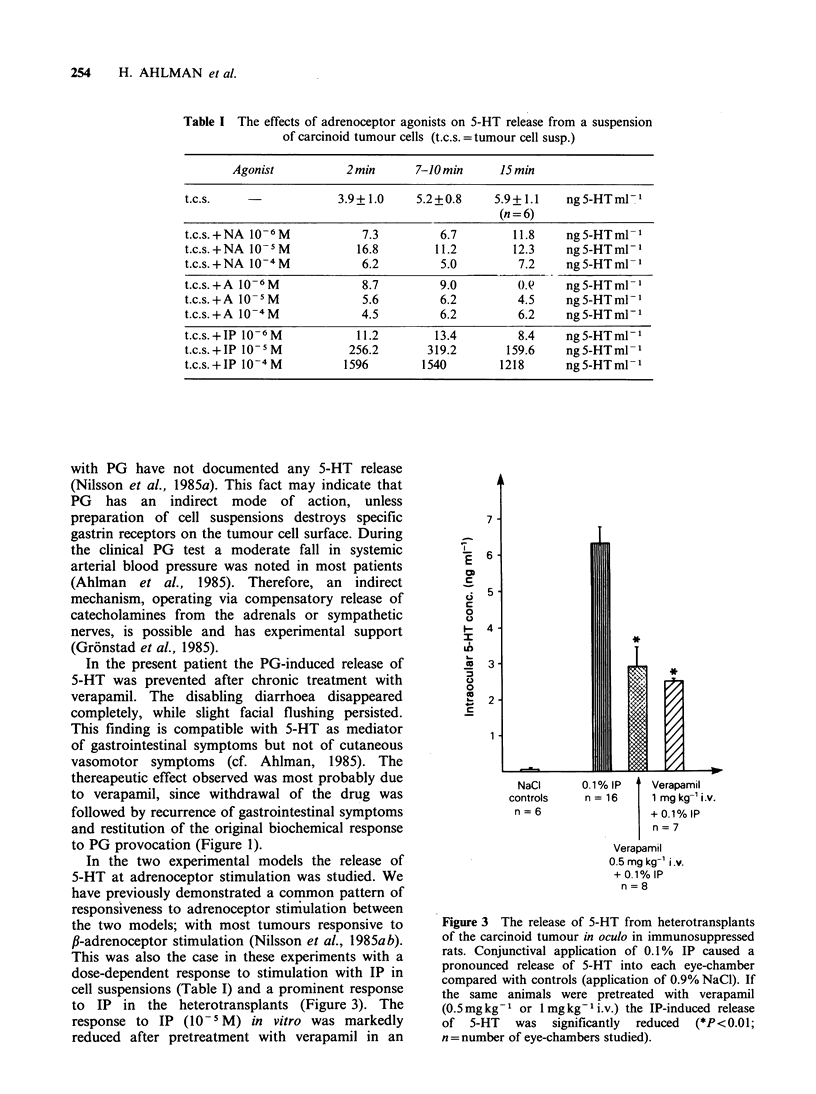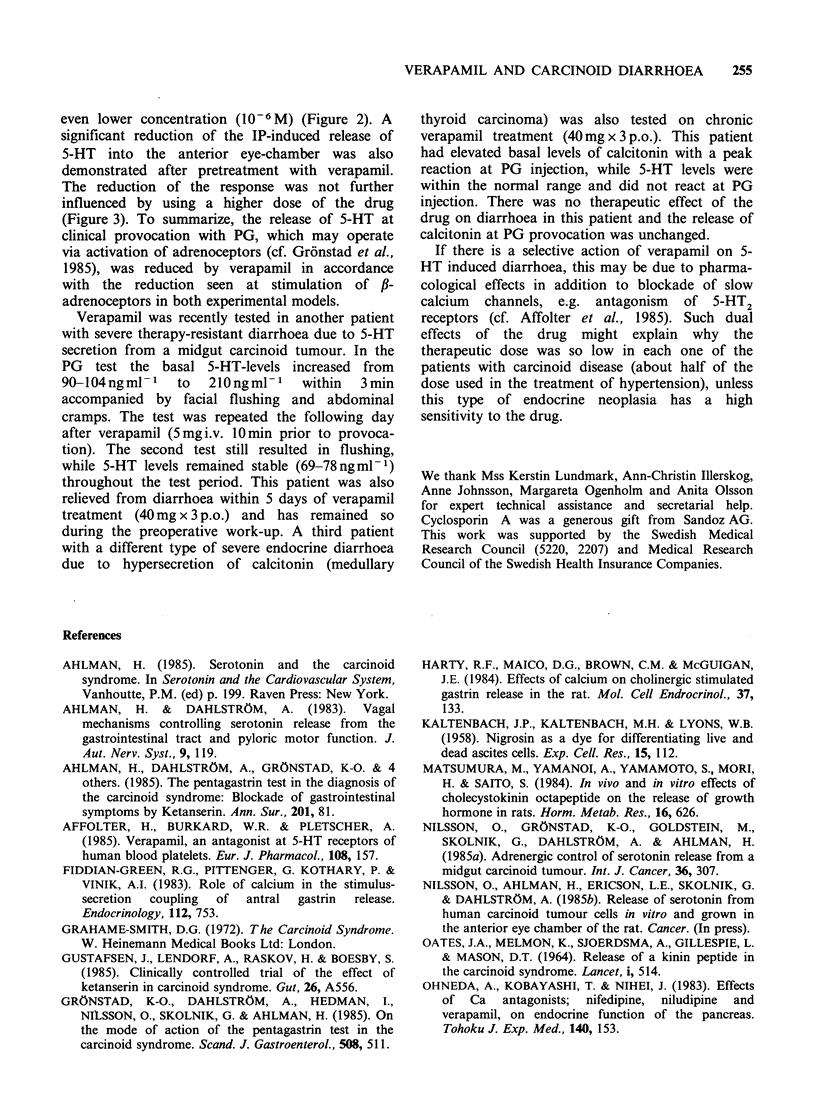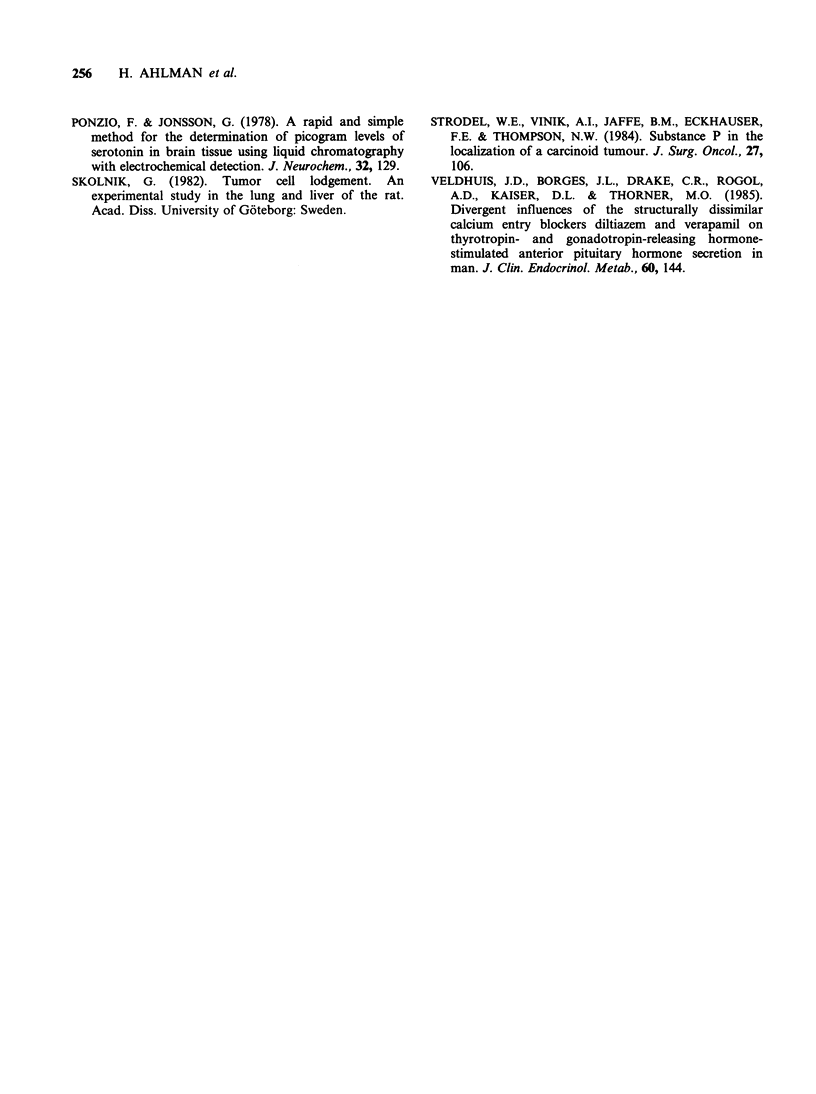# Verapamil and diarrhoea in the carcinoid syndrome--clinical and experimental observations on serotonin release.

**DOI:** 10.1038/bjc.1986.170

**Published:** 1986-08

**Authors:** H. Ahlman, O. Nilsson, K. O. Grönstad, G. Skolnik, L. E. Tisell, A. Dahlström

## Abstract

A patient with the midgut carcinoid syndrome with severe diarrhoea and proven hypersecretion of serotonin (5-HT) was treated with low doses of verapamil perorally. During treatment the patient was completely relieved of diarrhoea but discrete facial flushing persisted during treatment. When treatment was cessated, diarrhoeas recurred. This patient underwent pentagastrin (PG) provocation repeatedly; during untreated conditions injection of PG released 5-HT, detectable in peripheral venous blood. Such release was abolished during verapamil treatment, but recurred after withdrawal of the drug. Surgical biopsies from this tumour were studied in two experimental models: cell suspensions and heterotransplants grown in the anterior eye-chamber of immunosuppressed rats. Release of 5-HT from the cell suspensions was elicited in a dose-dependent manner after stimulation with isoprenaline (IP) suggesting activation of beta-adrenoceptors on the tumour cells. Such release was reduced after pretreatment with verapamil indicating a calcium dependent mechanism. Intraocular tumour transplants also responded with release of 5-HT into the chamber fluid after conjunctival application of IP. However, pretreatment of the rats with verapamil significantly reduced the IP-stimulated release of 5-HT.


*Br. J. Cancer* (1986), **54**, 251–256

### Verapamil and diarrhoea in the carcinoid syndrome—Clinical and experimental observations on serotonin release

H. Ahlman[1], O. Nilsson[2], K-O. Grönstad[1], G. Skolnik[1], L-E. Tisell[1] & A. Dahlström[2]

[1]*Department of Surgery I, Sahlgren Hospital, S-413 45 Göteborg, Sweden and* [2]*Institute of Neurobiology, University of Göteborg, Göteborg, Sweden.*

**Summary** A patient with the midgut carcinoid syndrome with severe diarrhoea and proven hypersecretion of serotonin (5-HT) was treated with low doses of verapamil perorally. During treatment the patient was completely relieved of diarrhoea but discrete facial flushing persisted during treatment. When treatment was cessated, diarrhoeas recurred. This patient underwent pentagastrin (PG) provocation repeatedly; during untreated conditions injection of PG released 5-HT, detectable in peripheral venous blood. Such release was abolished during verapamil treatment, but recurred after withdrawal of the drug. Surgical biopsies from this tumour were studied in two experimental models: cell suspensions and heterotransplants grown in the anterior eye-chamber of immunosuppressed rats. Release of 5-HT from the cell suspensions was elicited in a dose-dependent manner after stimulation with isoprenaline (IP) suggesting activation of $\beta$-adrenoceptors on the tumour cells. Such release was reduced after pretreatment with verapamil indicating a calcium dependent mechanism. Intraocular tumour transplants also responded with release of 5-HT into the chamber fluid after conjunctival application of IP. However, pretreatment of the rats with verapamil significantly reduced the IP-stimulated release of 5-HT.

Human carcinoid tumours of midgut origin produce and secrete large amounts of serotonin (5-HT). This monoamine has an established pathogenetic role in the development of diarrhoea and may also be involved in other symptoms of the carcinoid syndrome (cf. Grahame-Smith, 1972). 5-HT antagonists, i.e. methysergide or cyproheptadine, have been used with variable success in the treatment of carcinoid diarrhoea (cf. Ahlman, 1985). Selective antagonists of 5-HT$_2$ receptors, i.e. ketanserin, offered theoretically a more attractive therapeutic approach with less side effects. Although ketanserin was of therapeutic value in many patients with the carcinoid syndrome (Gustafsen *et al.*, 1985), a few patients had no symptomatic relief at all (Ahlman *et al.*, 1985). Such failure could be ascribed to secretion of compounds other than 5-HT by the tumour, e.g. bradykinin or substance P (Oates *et al.*, 1964, Strodel *et al.*, 1984).

In order to study the release of 5-HT from carcinoid tumour cells we have recently developed an experimental model with heterotransplantation of human carcinoid tumours into the anterior eye-chamber of immunosuppressed rats (Nilsson *et al.*, 1985*a*). The physiological release of 5-HT from gut enterochromaffin (EC) cells is controlled by $\beta$-adrenoceptors (Ahlman & Dahlström, 1983) and also carcinoid heterotransplants have an adrenoceptor controlled release mechanism (Nilsson *et al.*, 1985*a, b*). *In vitro* studies on tumour cell suspensions demonstrated that the release was blocked not only with $\beta$-adrenoceptor antagonists, but also with verapamil, a calcium channel blocker (Nilsson *et al.*, 1985*b*). This drug has previously been reported to block the release of several hormones under experimental conditions, e.g. from the anterior pituitary gland (Matsumura *et al.*, 1984; Veldhuis *et al.*, 1985), the endocrine pancreas (Ohneda *et al.*, 1983) and antral G-cells (Fiddian-Green *et al.*, 1983; Harty *et al.*, 1984).

The purpose of the present study was to evaluate the clinical effect of verapamil in the midgut carcinoid syndrome and the effect on provoked 5-HT release. Therefore we compared the release of 5-HT from cell suspensions and *in oculo* heterotransplants from the same tumour after adrenoceptor stimulation, and the influence of verapamil pretreatment in both systems was evaluated. Provocation of 5-HT release by pentagastrin (PG) was studied clinically in this patient before and during treatment with verapamil as well as after withdrawal of the drug.

### Materials and methods

### *Clinical history*

This patient (male, age 74) suffered from severe bouts of diarrhoea (daily frequency 10–15) and

Correspondence: H. Ahlman
Received 4 February 1986; and in revised form, 14 April 1986.

© The Macmillan Press Ltd., 1986

daily flushing since 3 months. Both facial flush and diarrhoea occurred regularly after the morning meal. From the preoperative investigation (CTscan, angiography) the patient was demonstrated to have an ileal tumour with large mesenteric lymphoglandular metastases and biliary spread to the liver. Urinary levels of 5-hydroxyindoleacetic acid were clearly elevated (12–18 $mmol\,mol^{-1}$ creatinine; ref. value 1–6). The patient had tried medical therapy (codeine, loperamide, diphenoxylate or ketanserin) for diarrhoea with little symptomatic relief. At surgery an ileal resection was performed as well as a debulking procedure directed against the large lymphogranular metastases in the mesenteric root.

*Tumour material*

The tumour had argyrophil and argentaffin staining properties and demonstrated a large proportion (>90%) of 5-HT immunoreactive cells. Fresh tumour material was obtained from the primary tumour, which was placed in ice-cooled, oxygenated Krebs' solution for 15–30 min prior to transplantation or preparation of tumour cell suspensions.

*Clinical provocation with PG*

Prior to surgery peripheral venous blood samples were drawn from the patient (fasting overnight) twice before (basal levels) and 1, 3 and 5 min after injection of PG (0.6 $\mu g\,kg^{-1}$ i.v.). Provocation with PG was performed (a) at admission to hospital (no medication), (b) after chronic peroral treatment with verapamil, (c) 1 week after cessation of verapamil. Since this drug is a widely used antihypertensive agent with few adverse effects, a clinical trial in patients with the carcinoid syndrome was recently approved by the Ethical Committee of the University of Göteborg.

Samples of whole blood (1 ml) were added to heparinized glass tubes containing 4 ml of distilled water (0.01% ascorbic acid added) and stored on ice. After complete haemolysis precipitation with 0.5 ml 1M NaOH and 1 ml 10% $ZnSO_4$ was performed. The samples were centrifuged at 200 *g* for 20 min and the sediments were discarded. In parallel samples, known amounts of 5-HT creatinine sulphate (25–100 pmol) were added to correct for recovery. Analyses were performed using Liquid Chromatography with Electro Chemical detection (LCEC) (cf. Ponzio & Jonsson, 1978). The supernatants were added to the columns in 10 $\mu l$ aliquots without further processing. Recovery of authentic 5-HT added averaged $72 \pm 5\%$. Standard curves were made by injecting standard solutions (2.5–10 pmol in 10 $\mu l$), prepared by dissolving 5-HT creatinine sulphate in 0.1 M perchloric acid.

*Tumour cell suspensions*

Fresh tumour material was thoroughly cleared of surrounding tissue, cut into small pieces and put into a 2% collagenase solution. Three hundred $\mu l$ of a 0.04% DNAse solution/10 ml collagenase solution was added to obtain a monodispersed cell suspension with maximal viability. The suspension was stirred for 60 min at room temperature under continuous oxygenation and thereafter filtered through a double layer of sterile gauze. The suspension was then centrifuged at 175 *g* for 5 min and washed and resuspended twice in Kreb's solution to remove the collagenase solution. The technique for preparation of tumour cell suspensions has been previously described in detail by Skolnik (1982). The cell viability in the final suspension was estimated after nigrosin staining according to Kaltenbach *et al.* (1958) and was 87%. The suspensions were diluted to a final concentration of $1 \times 10^6$ viable tumour cells $ml^{-1}$.

The cell suspensions were incubated at 37°C in oxygenated Kreb's solution alone (=control) or with noradrenaline (NA) ($10^{-6}$–$10^{-4}$ M), adrenaline (A) ($10^{-6}$–$10^{-4}$ M) or isoprenaline (IP) ($10^{-6}$–$10^{-4}$ M) added to the incubation medium. In all vials ascorbic acid (final concentration 0.01%) was added to the incubation medium to protect 5-HT and the adrenoceptor agonists from degradation. After 1–15 min of incubation, small samples (70 $\mu l$) were withdrawn and centrifuged at 10,000 *g* for 30–60 sec in an Airfuge (Beckman Inst. Inc.). The clear supernatant was decanted and immediately frozen at −20°C until 5-HT assay.

*Animals*

Male Sprague–Dawley rats (ALAB, Sollentuna, Sweden), weighing 100–150 g, were used as recipient animals for the transplantation experiments. Cyclosporin A (Sandoz AG, Basel, Switzerland) was injected s.c. (20 $mg\,kg^{-1}\,day^{-1}$) starting the day before transplantation. Animals were allowed free access to food and water and were kept in rooms with a 12 h dark/light cycle.

*In oculo transplantation*

Small tumour tissue pieces (1 $mm^3$) were dissected and manipulated to the lateral margin of each eye chamber by a technique previously described in detail (Nilsson *et al.*, 1985*a*). The transplants were rapidly vascularized (within 2–3 days) and retained the immunocytochemical profile of the original human tumour (Nilsson *et al.*, 1985*a,b*). After 7–10 days *in oculo* the transplants were pharmacologically stimulated via conjunctival application of solutions containing 0.1% NA, 0.1% A, 0.1% IP or 0.9% NaCl under light ether anaesthesia. After

15 min a small incision was made in the cornea and chamber fluid (5–10 μl) was withdrawn and assayed for 5-HT.

The experiments were repeated in the same rats after pretreatment with verapamil in two different doses (0.5 mg $kg^{-1}$ or 1.0 mg $kg^{-1}$ i.v.) 15 min prior to application of IP.

### Results

*Clinical provocation with PG*

At the initial PG test, basal 5-HT levels were not elevated, but within 1 min after PG injection the patient developed facial flushing and complained about abdominal cramps. Simultaneously peripheral 5-HT levels in whole blood were increased. The clinical and biochemical response declined after 5 min (Figure 1). The patient was thereafter treated with verapamil (Isoptin® 40 mg × 3 p.o.). After 2 weeks the patient complained about constipation and the dose was reduced to 40 mg × 2. After a total treatment period of 10 weeks PG provocation was again performed. The basal levels of 5-HT were now low and no peak was demonstrated after PG injection (Figure 1). The patient developed a mild facial flush but no gastrointestinal symptoms. To prove that the observed improvement was due to the drug therapy, verapamil treatment was suspended for 1 week. At the end of this period the patient had a few daily bouts of diarrhoea and PG provocation induced a similar clinical and biochemical response as in the untreated condition (Figure 1).

**Figure 1** Levels of 5-HT in peripheral venous whole blood at provocation with PG (0.6 μg $kg^{-1}$ i.v.) in a patient with the midgut carcinoid syndrome. The PG test was performed initially under untreated conditions (○). The PG-test was repeated after chronic treatment with verapamil perorally (●). After cessation of the drug for one week a third PG test was performed (△).

*Tumour cell suspensions*

The results of the stimulation experiments performed on carcinoid tumour cells in suspension are shown in Table I. The tumour cells released 5-HT into the incubation medium upon stimulation with IP ($10^{-5}-10^{-4}$ M) in a dose-dependent manner but not after stimulation with A or NA. Pretreatment with verapamil ($10^{-6}$ M) caused a pronounced reduction of the IP-induced ($10^{-5}$ M) 5-HT release (Figure 2). The release in this experimental group still exceeded the unstimulated tumour cell suspension studied during the same time interval (Figure 2).

**Figure 2** The release of 5-HT at stimulation of β-adrenoceptors (IP $10^{-5}$ M) in a tumour cell suspension (t.c.s.) was inhibited after pretreatment with verapamil ($10^{-6}$ M).

*In oculo experiments*

The results of the stimulation experiments performed in heterotransplants *in oculo* are summarized in Figure 3. Transplants in 8 animals released 5-HT into the chamber fluid upon application of IP in great excess of the 5-HT concentration observed in eyes with local saline application alone. The release was significantly reduced ($P<0.01$) after systemic pretreatment of the recipient animals with verapamil (0.5 mg $kg^{-1}$ i.v.). No further reduction was achieved by a higher dose of verapamil (1.0 mg $kg^{-1}$ i.v.).

### Discussion

The exact mechanism underlying the 5-HT response to PG has not been clarified. *In vitro* studies of carcinoid tumour cell suspensions after incubation

**Table I** The effects of adrenoceptor agonists on 5-HT release from a suspension of carcinoid tumour cells (t.c.s. = tumour cell susp.)

| *Agonist* | | *2 min* | *7–10 min* | *15 min* | |
|---|---|---|---|---|---|
| t.c.s. | — | $3.9 \pm 1.0$ | $5.2 \pm 0.8$ | $5.9 \pm 1.1$ ($n = 6$) | ng 5-HT $ml^{-1}$ |
| t.c.s. + NA $10^{-6}$ M | | 7.3 | 6.7 | 11.8 | ng 5-HT $ml^{-1}$ |
| t.c.s. + NA $10^{-5}$ M | | 16.8 | 11.2 | 12.3 | ng 5-HT $ml^{-1}$ |
| t.c.s. + NA $10^{-4}$ M | | 6.2 | 5.0 | 7.2 | ng 5-HT $ml^{-1}$ |
| t.c.s. + A $10^{-6}$ M | | 8.7 | 9.0 | 0.9 | ng 5-HT $ml^{-1}$ |
| t.c.s. + A $10^{-5}$ M | | 5.6 | 6.2 | 4.5 | ng 5-HT $ml^{-1}$ |
| t.c.s. + A $10^{-4}$ M | | 4.5 | 6.2 | 6.2 | ng 5-HT $ml^{-1}$ |
| t.c.s. + IP $10^{-6}$ M | | 11.2 | 13.4 | 8.4 | ng 5-HT $ml^{-1}$ |
| t.c.s. + IP $10^{-5}$ M | | 256.2 | 319.2 | 159.6 | ng 5-HT $ml^{-1}$ |
| t.c.s. + IP $10^{-4}$ M | | 1596 | 1540 | 1218 | ng 5-HT $ml^{-1}$ |

with PG have not documented any 5-HT release (Nilsson *et al.*, 1985*a*). This fact may indicate that PG has an indirect mode of action, unless preparation of cell suspensions destroys specific gastrin receptors on the tumour cell surface. During the clinical PG test a moderate fall in systemic arterial blood pressure was noted in most patients (Ahlman *et al.*, 1985). Therefore, an indirect mechanism, operating via compensatory release of catecholamines from the adrenals or sympathetic nerves, is possible and has experimental support (Grönstad *et al.*, 1985).

In the present patient the PG-induced release of 5-HT was prevented after chronic treatment with verapamil. The disabling diarrhoea disappeared completely, while slight facial flushing persisted. This finding is compatible with 5-HT as mediator of gastrointestinal symptoms but not of cutaneous vasomotor symptoms (cf. Ahlman, 1985). The thereapeutic effect observed was most probably due to verapamil, since withdrawal of the drug was followed by recurrence of gastrointestinal symptoms and restitution of the original biochemical response to PG provocation (Figure 1).

In the two experimental models the release of 5-HT at adrenoceptor stimulation was studied. We have previously demonstrated a common pattern of responsiveness to adrenoceptor stimulation between the two models; with most tumours responsive to $\beta$-adrenoceptor stimulation (Nilsson *et al.*, 1985*ab*). This was also the case in these experiments with a dose-dependent response to stimulation with IP in cell suspensions (Table I) and a prominent response to IP in the heterotransplants (Figure 3). The response to IP ($10^{-5}$ M) *in vitro* was markedly reduced after pretreatment with verapamil in an

**Figure 3** The release of 5-HT from heterotransplants of the carcinoid tumour *in oculo* in immunosuppressed rats. Conjunctival application of 0.1% IP caused a pronounced release of 5-HT into each eye-chamber compared with controls (application of 0.9% NaCl). If the same animals were pretreated with verapamil (0.5 mg $kg^{-1}$ or 1 mg $kg^{-1}$ i.v.) the IP-induced release of 5-HT was significantly reduced (*$P < 0.01$; $n$ = number of eye-chambers studied).

even lower concentration ($10^{-6}$ M) (Figure 2). A significant reduction of the IP-induced release of 5-HT into the anterior eye-chamber was also demonstrated after pretreatment with verapamil. The reduction of the response was not further influenced by using a higher dose of the drug (Figure 3). To summarize, the release of 5-HT at clinical provocation with PG, which may operate via activation of adrenoceptors (cf. Grönstad *et al.*, 1985), was reduced by verapamil in accordance with the reduction seen at stimulation of $\beta$-adrenoceptors in both experimental models.

Verapamil was recently tested in another patient with severe therapy-resistant diarrhoea due to 5-HT secretion from a midgut carcinoid tumour. In the PG test the basal 5-HT-levels increased from 90–104 ng ml$^{-1}$ to 210 ng ml$^{-1}$ within 3 min accompanied by facial flushing and abdominal cramps. The test was repeated the following day after verapamil (5 mg i.v. 10 min prior to provocation). The second test still resulted in flushing, while 5-HT levels remained stable (69–78 ng ml$^{-1}$) throughout the test period. This patient was also relieved from diarrhoea within 5 days of verapamil treatment (40 mg × 3 p.o.) and has remained so during the preoperative work-up. A third patient with a different type of severe endocrine diarrhoea due to hypersecretion of calcitonin (medullary thyroid carcinoma) was also tested on chronic verapamil treatment (40 mg × 3 p.o.). This patient had elevated basal levels of calcitonin with a peak reaction at PG injection, while 5-HT levels were within the normal range and did not react at PG injection. There was no therapeutic effect of the drug on diarrhoea in this patient and the release of calcitonin at PG provocation was unchanged.

If there is a selective action of verapamil on 5-HT induced diarrhoea, this may be due to pharmacological effects in addition to blockade of slow calcium channels, e.g. antagonism of 5-$HT_2$ receptors (cf. Affolter *et al.*, 1985). Such dual effects of the drug might explain why the therapeutic dose was so low in each one of the patients with carcinoid disease (about half of the dose used in the treatment of hypertension), unless this type of endocrine neoplasia has a high sensitivity to the drug.

We thank Mss Kerstin Lundmark, Ann-Christin Illerskog, Anne Johnsson, Margareta Ogenholm and Anita Olsson for expert technical assistance and secretarial help. Cyclosporin A was a generous gift from Sandoz AG. This work was supported by the Swedish Medical Research Council (5220, 2207) and Medical Research Council of the Swedish Health Insurance Companies.

### References

AHLMAN, H. (1985). Serotonin and the carcinoid syndrome. In *Serotonin and the Cardiovascular System*, Vanhoutte, P.M. (ed) p. 199. Raven Press: New York.

AHLMAN, H. & DAHLSTRÖM, A. (1983). Vagal mechanisms controlling serotonin release from the gastrointestinal tract and pyloric motor function. *J. Aut. Nerv. Syst.*, **9,** 119.

AHLMAN, H., DAHLSTRÖM, A., GRÖNSTAD, K-O. & 4 others. (1985). The pentagastrin test in the diagnosis of the carcinoid syndrome: Blockade of gastrointestinal symptoms by Ketanserin. *Ann. Sur.*, **201,** 81.

AFFOLTER, H., BURKARD, W.R. & PLETSCHER, A. (1985). Verapamil, an antagonist at 5-HT receptors of human blood platelets. *Eur. J. Pharmacol.*, **108,** 157.

FIDDIAN-GREEN, R.G., PITTENGER, G. KOTHARY, P. & VINIK, A.I. (1983). Role of calcium in the stimulus-secretion coupling of antral gastrin release. *Endocrinology*, **112,** 753.

GRAHAME-SMITH, D.G. (1972). *The Carcinoid Syndrome.* W. Heinemann Medical Books Ltd: London.

GUSTAFSEN, J., LENDORF, A., RASKOV, H. & BOESBY, S. (1985). Clinically controlled trial of the effect of ketanserin in carcinoid syndrome. *Gut*, **26,** A556.

GRÖNSTAD, K-O., DAHLSTRÖM, A., HEDMAN, I., NILSSON, O., SKOLNIK, G. & AHLMAN, H. (1985). On the mode of action of the pentagastrin test in the carcinoid syndrome. *Scand. J. Gastroenterol.*, **508,** 511.

HARTY, R.F., MAICO, D.G., BROWN, C.M. & McGUIGAN, J.E. (1984). Effects of calcium on cholinergic stimulated gastrin release in the rat. *Mol. Cell Endrocrinol.*, **37,** 133.

KALTENBACH, J.P., KALTENBACH, M.H. & LYONS, W.B. (1958). Nigrosin as a dye for differentiating live and dead ascites cells. *Exp. Cell. Res.*, **15,** 112.

MATSUMURA, M., YAMANOI, A., YAMAMOTO, S., MORI, H. & SAITO, S. (1984). *In vivo* and *in vitro* effects of cholecystokinin octapeptide on the release of growth hormone in rats. *Horm. Metab. Res.*, **16,** 626.

NILSSON, O., GRÖNSTAD, K-O., GOLDSTEIN, M., SKOLNIK, G., DAHLSTRÖM, A. & AHLMAN, H. (1985*a*). Adrenergic control of serotonin release from a midgut carcinoid tumour. *Int. J. Cancer*, **36,** 307.

NILSSON, O., AHLMAN, H., ERICSON, L.E., SKOLNIK, G. & DAHLSTRÖM, A. (1985*b*). Release of serotonin from human carcinoid tumour cells *in vitro* and grown in the anterior eye chamber of the rat. *Cancer*. (In press).

OATES, J.A., MELMON, K., SJOERDSMA, A., GILLESPIE, L. & MASON, D.T. (1964). Release of a kinin peptide in the carcinoid syndrome. *Lancet*, **i,** 514.

OHNEDA, A., KOBAYASHI, T. & NIHEI, J. (1983). Effects of Ca antagonists; nifedipine, niludipine and verapamil, on endocrine function of the pancreas. *Tohoku J. Exp. Med.*, **140,** 153.

PONZIO, F. & JONSSON, G. (1978). A rapid and simple method for the determination of picogram levels of serotonin in brain tissue using liquid chromatography with electrochemical detection. *J. Neurochem.*, **32,** 129.

SKOLNIK, G. (1982). Tumor cell lodgement. An experimental study in the lung and liver of the rat. Acad. Diss. University of Göteborg: Sweden.

STRODEL, W.E., VINIK, A.I., JAFFE, B.M., ECKHAUSER, F.E. & THOMPSON, N.W. (1984). Substance P in the localization of a carcinoid tumour. *J. Surg. Oncol.*, **27,** 106.

VELDHUIS, J.D., BORGES, J.L., DRAKE, C.R., ROGOL, A.D., KAISER, D.L. & THORNER, M.O. (1985). Divergent influences of the structurally dissimilar calcium entry blockers diltiazem and verapamil on thyrotropin- and gonadotropin-releasing hormone-stimulated anterior pituitary hormone secretion in man. *J. Clin. Endocrinol. Metab.*, **60,** 144.